# The Prognostic Role of Prothrombin Time and Activated Partial Thromboplastin Time in Patients with Newly Diagnosed Multiple Myeloma

**DOI:** 10.1155/2021/6689457

**Published:** 2021-05-19

**Authors:** Chuanying Geng, Guangzhong Yang, Huijuan Wang, Zhiyao Zhang, Huixing Zhou, Wenming Chen

**Affiliations:** Department of Hematology, Beijing Chaoyang Hospital, Capital Medical University, Beijing, China 100020

## Abstract

**Purpose:**

To evaluate the prognostic role of prothrombin time (PT) and activated partial thromboplastin time (APTT) for newly diagnosed multiple myeloma (MM).

**Methods:**

We retrospectively analyzed 354 patients with newly diagnosed MM who received primary treatment in our center. The propensity score matching technique was used to reduce the bias between groups.

**Results:**

Among 354 patients, lengthened PT or APTT was observed in 154 (43.5%) patients and 200 (56.5%) patients had normal PT and APTT. Patients with lengthened PT or APTT had significantly shorter median overall survival (OS) (37.5 vs. 73.8 months, *p* < 0.001) and progression-free survival (PFS) (23.1 vs. 31.6 months, *p* = 0.001) than those with normal PT and APTT. Univariate Cox proportional hazards regression analyses showed that lengthened PT or APTT was associated with shorter OS (HR = 2.100, 95% CI: 1.525-2.893, *p* < 0.001). Lengthened PT or APTT was also a poor prognostic factor for OS (HR = 3.183, 95% CI: 1.803-5.617, *p* < 0.001) in multivariable analyses. The poor effect of lengthened PT or APTT on PFS was confirmed in univariate analysis (HR = 1.715, 95% CI: 1.244-2.365, *p* = 0.001), but it had no impact on PFS in multivariate analysis (*p* = 0.197). In the propensity score matching analysis, 154 patients, 77 in each group, were identified. Among 154 matched patients, the OS of patients with lengthened PT or APTT was shorter (38.4 vs. 51.0 months, *p* = 0.030), but PFS was similar (29.0 vs. 35.0 months, *p* = 0.248).

**Conclusion:**

These results demonstrated that lengthened PT or APTT was an independent poor prognostic factor for patients with newly diagnosed MM.

## 1. Introduction

Multiple myeloma (MM) is the second most common hematologic malignancy which originates from the proliferation of clonal plasma cells [1]. MM mainly presents with hypercalcemia, *renal* insufficiency, anemia, and bone lesions [2, 3]. Patients with MM also commonly presented abnormal prothrombin time (PT), activated partial thromboplastin time (APTT), and thrombin time [4]. In the 1970s, Sanchez-Avalos et al. [5] investigated the coagulation mechanism in 34 patients with MM and found lengthened PT and TT. Moreover, the ratio of lengthened PT was different in patients with IgG, IgA, and IgM [6, 7]. There were 5-86% patients with MM presenting abnormal PT and 8.9-69% patients with abnormal APTT [4, 8–11]. Half of the patients with MM had a lengthened PT, but few patients displayed bleeding diathesis [12]. Patients with lengthened PT had high level of monoclonal proteins which were related to PT [11, 13]. One study showed that lengthened APTT was an independent prognostic factor in patients with MM [14]. However, one study reported that lengthened PT and APTT were not poor prognostic factors for survival in MM patients [15]. At present, the prognostic role of PT and APTT in MM is still controversial.

We retrospectively analyzed PT and APTT status in newly diagnosed MM patients who received initial therapy in our center. The aim of this study was to evaluate the prognostic role of PT and APTT in newly diagnosed MM patients.

## 2. Methods

### 2.1. Patients

We recorded the baseline data of patients with newly diagnosed MM who received treatment in our center between October 1, 2008, and October 1, 2019. All patients were diagnosed according to the definition of multiple myeloma of the International Myeloma Working Group and followed up until May 1, 2020 [3]. Patients received measurement of PT and APTT before antimyeloma therapy. The reference range of the PT was 9.6-13.0 seconds, and that of the APTT was 21.0-34.0 seconds. Immunoglobulins IgG, IgA, and IgM were also detected by standard nephelometry. Deep immunoparesis was defined as one of uninvolved immunoglobulins below 50% normal levels, and full immunoparesis was defined as at least two suppressed uninvolved immunoglobulins. The fluorescence in situ hybridization (FISH) assay was used to detect cytogenetic abnormalities including del17p13, t(14; 16), and t(4; 14). Approval for this study was obtained from the medical ethics committee at Beijing Chaoyang Hospital. The informed consent of patients was provided in accordance with the Declaration of Helsinki.

### 2.2. Response and Outcome Measures

The IMWG criteria were used to evaluate the treatment effect [16]. Progression-free survival (PFS) was estimated from the time of diagnosis to disease progression or death, and overall survival (OS) from the time of diagnosis to death of any cause or the date of the last follow-up. PFS and OS were the primary endpoints. Patients will be censored if they could not be contacted at the last follow-up day.

### 2.3. Statistical Analysis

Categorical variables were analyzed by the method of the chi-square test or Fisher's exact test. The Kaplan-Meier survival curves and the log-rank test were used to compare OS and PFS of different groups. The prognostic impact was evaluated by the Cox proportional hazards regression analyses, and results were reported as hazard ratios (HRs) with 95% confidence intervals (95% CIs). To balance the distribution of factors which had prognostic value in previous studies or had prognostic value according to results of multivariate Cox proportional hazards regression analyses in this study, we used a propensity score matching technique to control differences in baseline characteristics. The SPSS 23.0 software (SPSS Institute) was used for statistical analyses. A *p* value < 0.05 indicated statistical significance, and all tests were two-sided.

## 3. Results

### 3.1. Patient Characteristics

**Figure 1:**
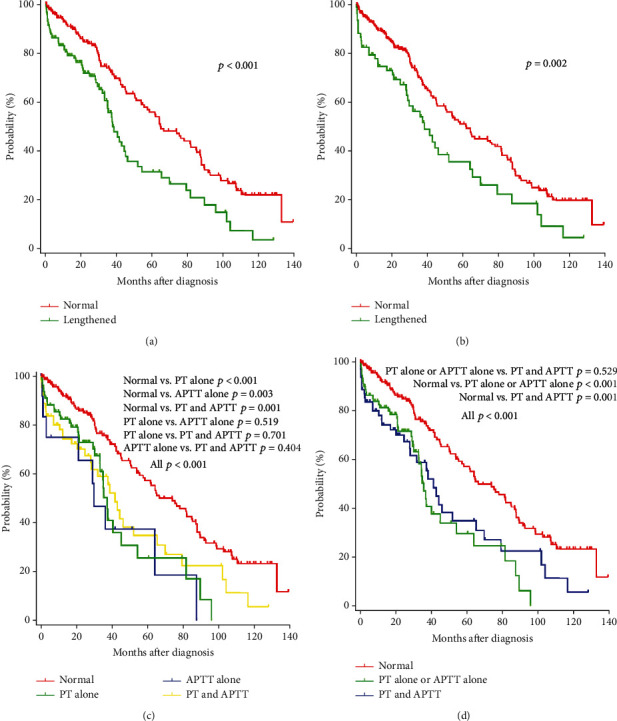
Kaplan-Meier survival curves on OS of patients with newly diagnosed MM: (a) PT; (b) APTT; (c) PT and APTT; (d) PT and APTT.

A total of 354 patients with newly diagnosed MM were analyzed. Lengthened PT was observed in 39.8% (141/354) patients and lengthened APTT in 21.2% (75/354) patients. There were 22.3% (79/354) patients with lengthened PT alone, 3.7% (13/354) patients with lengthened APTT alone, 17.5% (62/354) patients with lengthened PT and APTT, and 56.5% (200/354) patients with normal PT and APTT. Patients with normal PT and APTT had significantly longer OS than those with lengthened PT alone (*p* < 0.001), lengthened APTT alone (*p* = 0.003), and lengthened PT and APTT (*p* = 0.001) (Figure 1(c)). Moreover, there was no difference in OS among these three groups (*p* > 0.05, Figure 1(c)). For evaluating the effect of PT and APTT on survival of patients with newly diagnosed MM, we divided patients into two groups: the normal group had 200 patients with normal PT and APTT and the lengthened group had 154 patients with lengthened PT or APTT.  Table 1 shows the characteristics of 354 patients. The median age was 61 (35–87) years, and 55.4% patients were male. The most common monoclonal protein was the IgG type (44.9%). Among 354 patients, 172 (48.6%) were at International Scoring System (ISS) stage III, and 62 (19.7%) were at revised ISS (R-ISS) stage III. All patients received induction therapy combining novel drugs: 144 (43.5%) patients received therapy combining bortezomib, 50 (15.1%) combining immunomodulatory (IMiD), and 137 (41.4%) combining bortezomib and IMiD. After induction therapy, 78 (22.7%) patients received autologous stem cell transplant (ASCT).  Table 1 shows that there were statistically significant differences between normal and lengthened groups in the MM subtype, serum albumin, serum *β*2-microglobulin (*β*2-MG), hemoglobin, and platelet.

### 3.2. Multivariate Analysis for Survival

Univariate Cox proportional hazards regression analyses determined eleven factors associated with OS: age > 65years, *β*2‐MG ≥ 3.5 mg/L, serum albumin ≥ 35 g/L, serum creatinine ≥ 88.4 *μ*mol/L, serum calcium > 2.75 mmol/L, lactate dehydrogenase (LDH) ≥ 250 U/L, deep and full immunoparesis, lengthened PT or APTT, platelet ≥ 100 × 10^9^/L, light chain *λ* type, and ASCT (Table 2). Multivariate analysis was performed for these eleven covariates, del(17p13), t(14; 16), and t(4; 14). It was shown that lengthened PT or APTT was independently related to shorter OS (HR = 3.183, 95% CI: 1.803-5.617, *p* < 0.001). Other factors that were independently associated with OS in the multivariate analysis included age > 65years, serum calcium > 2.75 mmol/L, LDH ≥ 250 U/L, deep and full immunoparesis, light chain *λ* type, del(17p13), and ASCT (Table 2). A multivariate analysis for PFS was performed in the same models and showed that lengthened PT or APTT was not significantly associated with PFS (*p* = 0.197, Supplementary Table 1).

### 3.3. Matched Pairs of Patients

Patients with lengthened PT or APTT and with normal PT and APTT were matched for age, hemoglobin, albumin, LDH, creatinine, calcium, *β*2-MG, platelet, deep and full immunoparesis, light chain *λ* type, del(17p13), t(14; 16), t(4; 14), and ASCT. Among 354 patients, 154 patients, 77 in each group, were identified by the propensity score matching technique. It showed that matched groups were not significantly different with respect to any characteristics (Table 3).

### 3.4. Response

Of the 324 patients who could be evaluated to have the best response after induction therapy, 280 (86.4%) patients achieved partial response (PR) or better after induction treatment. Sixty-four patients (19.8%) achieved stringent complete response (sCR), 53 (16.4%) achieved complete response (CR), 72 (22.2%) achieved very good partial response (VGPR), and 91 (28.1%) achieved PR. It showed that lengthened PT or APTT had no impact on the best response compared with the normal group (*p* = 0.090, Supplementary Table 2). Of the 154 matched patients, 129 (83.8%) patients achieved PR or better after induction treatment. Thirty-three patients (21.4%) achieved sCR, 24 (15.6%) achieved CR, 37 (24.0%) achieved VGPR, and 35 (22.7%) achieved PR. Patients with lengthened PT or APTT had also no impact on response compared with normal patients (*p* = 0.475, Supplementary Table 3).

### 3.5. Survival

**Figure 2:**
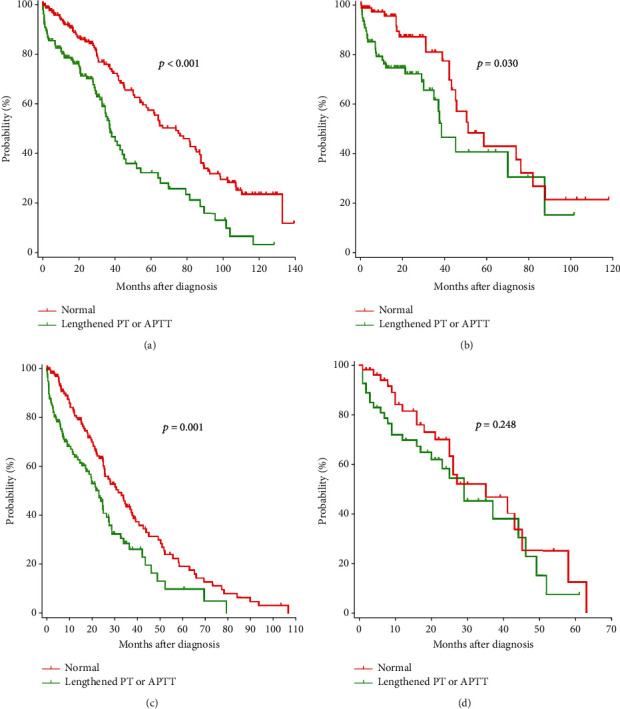
Kaplan-Meier survival curves on OS and PFS of patients with newly diagnosed MM: (a) OS for all patients; (b) OS for matched patients; (c) PFS for all patients; (d) PFS for matched patients.

The median follow-up time for all patients was 24.0 (range 0.2-139.2) months. The median OS estimated by the Kaplan-Meier method were 37.5 (95% CI, 32.4-42.6) and 73.8 (95% CI, 56.4-91.2) months for all patients with lengthened PT or APTT and with normal PT and APTT, respectively (*p* < 0.001, Figure 2(a)). The median PFS estimated were 23.1 (95% CI, 18.5-27.7) and 31.6 (95% CI, 24.1-39.1) months for all patients with lengthened PT or APTT and with normal PT and APTT, respectively (*p* = 0.007, Figure 2(c)). The median OS were 38.4 (95% CI, 29.0-47.8) and 51.0 (95% CI, 33.7-68.3) months for matched patients with lengthened PT or APTT and with normal PT and APTT, respectively (*p* = 0.030, Figure 2(b)). The median PFS were 29.0 (95% CI, 17.1-40.9) and 35.0 (95% CI, 17.4-52.6) months for matched patients with lengthened PT or APTT and with normal PT and APTT, respectively (*p* = 0.248, Figure 2(d)).

## 4. Discussion

In this study, we assessed the prognostic impact of lengthened PT and APTT on the outcome of patients with newly diagnosed MM. Lengthened PT was observed in 39.8% patients and lengthened APTT in 21.2% patients. There were 22.3% patients with lengthened PT alone, 3.7% patients with lengthened APTT alone, 17.5% patients with lengthened PT and APTT, and 56.5% patients with normal PT and APTT. We found that PT or APTT was an independent poor prognostic factor for OS in patients with newly diagnosed MM; however, it had no impact on PFS. The propensity score matching analysis also showed that patients with lengthened PT or APTT had shorter OS, but similar PFS. Patients with lengthened PT or APTT had a similar response rate compared with patients without normal PT and APTT.

Several studies showed that 5-86% patients with MM presented abnormal PT and 8.9-69% patients presented abnormal APTT [4, 8–11]. Elice et al. [10] found that 24% patients with newly diagnosed MM presented abnormal PT (normal range: 11.5-14.7 s) and 11% patients with newly diagnosed MM presented abnormal APTT (normal range: 23.0-36.9 s). Huang et al. [11] retrospectively analyzed 101 patients with newly diagnosed multiple myeloma and reported that lengthened APTT (28.5–41.5 s) was observed in 9 (8.9%) patients and lengthened PT (11.5–14.5 s) in 5 (5%) patients. Gogia et al. [9] analyzed 29 MM patients and 30 age-matched controls and showed that lengthened PT and APTT were observed in 14 (48.3%) and 20 (69%) patients, respectively; both PT and APTT were lengthened in 10 (34.5%) patients. Pandey et al. [13] found that 52 (33.1%) patients presented isolated lengthened PT which was more often detected in MM patients compared to other plasma cell disorders. Kyle et al. [8] analyzed 1027 patients with newly diagnosed multiple myeloma and found that 37% patients presented lengthened PT (>12 s) among 370 patients who received a test. Our study showed that lengthened PT was observed in 39.8% patients that was similar to the two large studies [8, 13]. Patients have significantly different lengthened APTT ranging from 8.9% to 69% [9–11]. Our study showed that 21.2% patients presented lengthened APTT which was less than lengthened PT. In this study, 17.5% patients had both lengthened PT and APTT that were less than those of the previous study [9]. Few studies had evaluated the incidence of lengthened PT and APTT, and it needs more large studies to confirm its incidence in patients with newly diagnosed MM.

The prognostic role of lengthened PT and APTT in newly diagnosed MM has been evaluated in several studies. Teng et al. [14] analyzed 222 patients with newly diagnosed MM and found that lengthened APTT was an independent prognostic factor; however, lengthened PT had no effect. Moreover, it showed that lengthened APTT was a poor factor for survival of patients with IgA-type MM, but not for IgG MM patients. However, Shen et al. [15] retrospectively analyzed 65 MM patients with bone lesions who need surgical treatment and found that PT and APTT were not risk factors for survival in multivariate Cox regression analysis. At present, few studies reported the impact of PT and APTT on the outcome of patients with newly diagnosed MM. In this study, patients with lengthened PT had significant shorter OS estimated by the Kaplan-Meier method than those with normal PT (Figure 1(a)). Patients with lengthened APTT also had significant shorter OS (Figure 1(b)). Patients with normal PT and APTT had significantly longer OS than those with lengthened PT alone, lengthened APTT alone, and lengthened PT and APTT. Moreover, there was no difference in OS among these three groups. So, we divided patients into two groups: the normal group had 200 (56.5%) patients with normal PT and APTT and the lengthened group had 154 (43.5%) patients with lengthened PT or APTT. We showed that patients with lengthened PT or APTT had significant shorter OS and PFS estimated by the Kaplan-Meier method than those with normal PT and APTT. Multivariate analysis revealed that lengthened PT or APTT was an independent poor prognostic factor for OS of patients with newly diagnosed MM. However, lengthened PT or APTT was not significantly associated with PFS. Moreover, we used propensity score matching analysis to balance covariate distributions between patients with lengthened PT or APTT and with normal PT and APTT. Patients with lengthened PT or APTT had significant shorter OS compared with patients with normal PT and APTT after balancing the main factors. Propensity score matching analysis showed that PFS was similar between the two groups. We also showed that lengthened PT or APTT had no impact on response to primary therapy. As a result, lengthened PT or APTT may be considered an independent poor prognostic factor.

There are some other laboratory biomarkers which are economical and clinical available prognostic factors for newly diagnosed MM. Complete blood count is one of the most inexpensive tests required during MM diagnosis and treatment. Yang et al. [17] measured absolute lymphocyte count (ALC) and absolute monocyte count in routine blood samples from 102 patients with newly diagnosed MM and found that ALC and the lymphocyte-monocyte ratio (LMR) could serve as prognostic markers for patients with newly diagnosed MM. However, another study found that ALC was not related to prognosis of newly diagnosed MM patients [18]. Gu et al. [19] reported that T lymphocyte subsets were crucial prognostic factors for newly diagnosed MM and low CD4+ T cell counts and the CD4/CD8 ratio were independent poor prognostic markers. The neutrophil-to-lymphocyte ratio (NLR) and platelet-to-lymphocyte ratio (PLR) also had a significant independent prognostic value for multiple myeloma patients [20]. Other routine tests, such as circulating plasma cells, renal function, normal immunoglobulins level, and serum calcium, were also considered valuable prognostic factors for newly diagnosed MM [21–24]. Some routine and inexpensive clinical tests, such as PT and APTT, may have useful prognostic value for patients with newly diagnosed MM.

This study has some limitations. The database was collected from a multiple myeloma center, and the number of patients in this study was relatively small. These patients were all from northern China, and the findings may be more applicable to Chinese patients. This study was a retrospective analysis in nature which may cause bias. It was a large MM center in Beijing, and some data of patients may refer from other medical centers resulting in missing some data. Because of economic constraints, some patients did not receive tests of cytogenetic abnormalities by FISH. This may interfere to evaluate the prognostic value of PT and APTT. Finally, the follow-up time was short, and it needs larger population to confirm these results.

## 5. Conclusion

Our study demonstrated that lengthened PT or APTT is a poor prognostic factor for patients with newly diagnosed MM. It needs further study to verify the prognostic role of PT and APTT in the future.

## Figures and Tables

**Table 1:** Baseline clinical and biological characteristics of MM patients.

Characteristics	All patients	Normal PT and APTT	Lengthened PT or APTT	*p* value
	*n* = 354	*n* = 200	*n* = 154	
	*n* (%)	*n* (%)	*n* (%)	
Sex				
Male	196 (55.4)	113 (56.5)	83 (53.9)	0.625
Female	158 (44.6)	87 (43.5)	71 (46.1)	
Age				
≤65 years	237 (66.9)	138 (69.0)	99 (64.3)	0.350
>65 years	117 (33.1)	62 (31.0)	55 (35.7)	
MM subtype				
IgG	159 (44.9)	80 (40.0)	79 (51.3)	0.000
IgA	83 (23.4)	34 (17.0)	49 (31.8)	
IgD	17 (4.8)	11 (5.5)	6 (3.9)	
Light chain only	85 (24.0)	69 (34.5)	16 (10.4)	
Nonsecretory	10 (2.8)	6 (3.0)	4 (2.6)	
Light chain				
*κ*	173 (50.3)	102 (52.6)	71 (47.3)	0.335
*λ*	171 (49.7)	92 (47.4)	79 (52.7)	
ISS stage				
I	46 (13.0)	32 (16.0)	14 (9.1)	0.086
II	136 (38.4)	79 (39.5)	57 (37.0)	
III	172 (48.6)	89 (44.5)	83 (53.9)	
R-ISS stage				
I	32 (10.2)	21 (12.2)	11 (7.7)	0.139
II	220 (70.1)	123 (71.5)	97 (68.3)	
III	62 (19.7)	28 (16.3)	34 (23.9)	
Serum albumin				
<35 g/L	200 (56.7)	91 (45.7)	109 (70.8)	0.000
≥35 g/L	153 (43.3)	108 (54.3)	45 (29.2)	
Serum *β*2-microglobulin				
<3.5 mg/L	98 (29.9)	66 (36.1)	32 (22.1)	0.006
≥3.5 mg/L	230 (70.1)	117 (63.9)	113 (77.9)	
Hemoglobin				
<100 g/L	235 (66.6)	113 (56.5)	122 (79.7)	0.000
≥100 g/L	118 (33.4)	87 (43.5)	31 (20.3)	
Platelet				
<100 × 10^9^/L	53 (15.0)	21 (10.5)	32 (20.9)	0.007
≥100 × 10^9^/L	300 (85.0)	179 (89.5)	121 (79.1)	
Serum creatinine				
<88.4 *μ*mol/L	200 (56.7)	119 (59.8)	81 (52.6)	0.176
≥88.4 *μ*mol/L	153 (43.3)	80 (40.2)	73 (47.4)	
Corrected serum calcium				
≤2.75 mmol/L	308 (87.3)	179 (89.9)	129 (83.8)	0.084
>2.75 mmol/L	45 (12.7)	20 (10.1)	25 (16.2)	
Lactate dehydrogenase				
<250 U/L	298 (84.9)	170 (85.9)	128 (83.7)	0.568
≥250 U/L	53 (15.1)	28 (14.1)	25 (16.3)	
Deep and full immunoparesis				
Yes	106 (41.9)	53 (40.2)	53 (43.8)	0.557
No	147 (58.1)	79 (59.8)	68 (56.2)	
Cytogenetic abnormalities by FISH				
del(17p13)				
Abnormality	22 (9.0)	9 (7.0)	13 (11.2)	0.255
No abnormality	222 (91.0)	119 (93.0)	103 (88.8)	
t(14; 16)				
Abnormality	5 (2.0)	2 (1.6)	3 (2.6)	0.573
No abnormality	239 (98.0)	126 (98.4)	113 (97.4)	
t(4; 14)				
Abnormality	41 (16.8)	22 (17.2)	19 (16.4)	0.866
No abnormality	203 (83.2)	106 (82.8)	97 (83.6)	
Induction regimes				
Bortezomib based	144 (43.5)	75 (39.3)	69 (49.3)	0.192
IMiD based	50 (15.1)	31 (16.2)	19 (13.6)	
Bortezomib and IMiD based	137 (41.4)	85 (44.5)	52 (37.1)	
ASCT				
Yes	78 (22.7)	47 (24.0)	31 (20.9)	0.506
No	266 (77.3)	149 (76.0)	117 (79.1)	

Abbreviations: PT: prothrombin time; APTT: activated partial thromboplastin time; IMiD: immunomodulatory; ASCT: autologous stem cell transplant.

**Table 2:** Cox analysis (univariate and multivariate) of prognostic factors for OS.

	Univariate			Multivariate		
	HR	95% CI	*p* value	HR	95% CI	*p* value
Age > 65 years	1.477	1.063-2.054	0.020	1.737	0.982-3.073	0.058
*β*2‐microglobulin ≥ 3.5 mg/L	1.608	1.116-2.315	0.011			
Albumin ≥ 35 g/L	0.589	0.417-0.832	0.003			
Hemoglobin ≥ 100 g/L	0.688	0.492-0.963	0.029			
Creatinine ≥ 88.4 *μ*mol/L	1.547	1.133-2.114	0.006			
Calcium > 2.75 mmol/L	1.650	1.076-2.530	0.022	2.354	1.173-4.724	0.016
Lactate dehydrogenase ≥ 250 U/L	2.749	1.787-4.229	0.000	1.890	0.991-3.606	0.053
Deep and full immunoparesis	1.734	1.124-2.676	0.013	2.298	1.351-3.909	0.002
Lengthened PT or APTT	2.100	1.525 2.893	0.000	3.183	1.803-5.617	0.000
Platelet ≥ 100 × 10^9^/L	0.518	0.344-0.781	0.002			
Light chain *λ* type	1.813	1.314-2.504	0.000	1.672	0.967-2.890	0.066
Induction regimes						
Bortezomib based			0.421			
IMiD based			0.610			
Bortezomib and IMiD based			0.194			
del(17p13)			0.054	2.296	1.007-5.235	0.048
t(14; 16)			0.833			
t(4; 14)			0.071			
ASCT	0.404	0.264-0.617	0.000	0.396	0.183-0.859	0.019

Abbreviations: PT: prothrombin time; APTT: activated partial thromboplastin time; IMiD: immunomodulatory; ASCT: autologous stem cell transplant.

**Table 3:** Characteristics between matched patients with normal and prolonged PT or APTT.

Characteristics	Normal PT and APTT	Lengthened PT and APTT	*p* value
	*n* = 77	*n* = 77	
	*n* (%)	*n* (%)	
Age			
≤65 years	48 (62.3)	52 (67.5)	0.499
>65 years	29 (37.7)	25 (32.5)	
Light chain			
*κ*	40 (51.9)	43 (55.8)	0.628
*λ*	37 (48.1)	34 (44.2)	
ISS stage			
I	10 (13.0)	13 (16.9)	0.638
II	30 (39.0)	25 (32.5)	
III	37 (48.1)	39 (50.6)	
R-ISS stage			
I	9 (11.7)	10 (13.0)	0.876
II	53 (68.8)	50 (64.9)	
III	15 (19.5)	17 (22.1)	
Serum albumin			
<35 g/L	40 (51.9)	39 (50.6)	0.872
≥35 g/L	37 (48.1)	38 (49.4)	
Serum *β*2-microglobulin			
<3.5 mg/L	22 (28.6)	21 (27.3)	0.857
≥3.5 mg/L	55 (71.4)	56 (72.7)	
Hemoglobin			
<100 g/L	57 (74.0)	54 (70.1)	0.590
≥100 g/L	20 (26.0)	23 (29.9)	
Platelet			
<100 × 10^9^/L	13 (16.9)	9 (11.7)	0.357
≥100 × 10^9^/L	64 (83.1)	68 (88.3)	
Serum creatinine			
<88.4 *μ*mol/L	45 (58.4)	44 (57.1)	0.870
≥88.4 *μ*mol/L	32 (41.6)	33 (42.9)	
Corrected serum calcium			
≤2.75 mmol/L	66 (85.7)	66 (85.7)	1.000
>2.75 mmol/L	11 (14.3)	11 (14.3)	
Lactate dehydrogenase			
<250 U/L	64 (83.1)	66 (85.7)	0.657
≥250 U/L	13 (16.9)	11 (14.3)	
Deep and full immunoparesis			
Yes	33 (42.9)	36 (46.8)	0.627
No	44 (57.1)	41 (53.2)	
Cytogenetic abnormalities by FISH			
del(17p13)			
Abnormality	8 (10.4)	8 (10.4)	1.000
No abnormality	69 (89.6)	69 (89.6)	
t(14; 16)			
Abnormality	0 (0.0)	1 (1.3)	0.316
No abnormality	77 (100.0)	76 (98.7)	
t(4; 14)		
Abnormality	11 (14.3)	14 (18.2)	0.512
No abnormality	66 (85.7)	63 (81.8)	
Induction regimes		
Bortezomib based	39 (50.6)	44 (57.1)	0.636
IMiD based	10 (13.0)	7 (9.1)	
Bortezomib and IMiD based	28 (36.4)	26 (33.8)	
ASCT		
Yes	16 (20.8)	17 (22.1)	0.844
No	61 (79.2)	60 (77.9)	

Abbreviations: PT: prothrombin time; APTT: activated partial thromboplastin time; IMiD: immunomodulatory; ASCT: autologous stem cell transplant.

## Data Availability

The analyzed datasets generated during the study are available from the corresponding author on reasonable request.

## Supplementary Materials

Supplementary Materials Supplementary Material 1: univariate Cox proportional hazards regression analyses showed that lengthened PT or APTT was a poor prognostic factor for PFS of newly diagnosed MM patients, but multivariate analysis showed that it was not significantly associated with PFS. Supplementary Material 2: the best response rate after induction therapy in all patients. Supplementary Material 3: the best response rate after induction therapy in matched patients.
