# Supplementary material for: The Prognostic Role of Prothrombin Time and Activated Partial Thromboplastin Time in Patients with Newly Diagnosed Multiple Myeloma
Source: Biomed Res Int. 2021 May 19;2021:6689457. doi: 10.1155/2021/6689457 (PMC8159641; doi:10.1155/2021/6689457)
Supplement: Supplementary Materials — Supplementary Material 1: univariate Cox proportional hazards regression analyses showed that lengthened PT or APTT was a poor prognostic factor for PFS of newly diagnosed MM patients, but multivariate analysis showed that it was not significantly associated with PFS. Supplementary Material 2: the best response rate after induction therapy in all patients. Supplementary Material 3: the best response rate after induction therapy in matched patients. [file 6689457.f1.docx]

| Supplementary table 1 . Cox analysis (univariate and multivariate) of prognostic factors for PFS | | | | | | | |
| --- | --- | --- | --- | --- | --- | --- | --- |
|  | | | | | | | |
|  | Univariate | | | Multivariate | | | |
|  | HR | 95% CI | *p* value |  | HR | 95% CI | *p* value |
| Age >65years | 1.522 | 1.078-2.147 | 0.017 |  |  |  |  |
| β2 microglobulin ≥3.5mg/L | 1.442 | 1.004-2.072 | 0.048 |  |  |  |  |
| Albumin ≥35g/L | 0.635 | 0.453-0.890 | 0.008 |  |  |  |  |
| Haemoglobin ≥100g/L | 0.660 | 0.469-0.929 | 0.017 |  |  |  |  |
| Creatinine ≥88.4μmol/L |  |  | 0.136 |  |  |  |  |
| Calcium >2.75mmol/L |  |  | 0.210 |  |  |  |  |
| Lactate dehydrogenase≥250U/L | 2.197 | 1.425-3.388 | 0.000 |  | 1.937 | 1.050-3.571 | 0.034 |
| Deep and full immunoparesis | 0.604 | 0.399-0.916 | 0.018 |  | 1.765 | 1.052-2.960 | 0.031 |
| Lengthened PT or APTT | 1.715 | 1.244-2.365 | 0.001 |  |  |  |  |
| Platelet ≥100x10^9^/L | 0.343 | 0.228-0.518 | 0.000 |  | 0.456 | 0.236-0.880 | 0.019 |
| Light chain λ type |  |  | 0.464 |  |  |  |  |
| Induction regimes |  |  | 0.357 |  |  |  |  |
| Bortezomib based |  |  | 0.590 |  |  |  |  |
| IMiD based |  |  | 0.307 |  |  |  |  |
| Bortezomib and IMiD based |  |  | 0.303 |  |  |  |  |
| del(17p13) |  |  | 0.690 |  |  |  |  |
| t(14; 16) |  |  | 0.743 |  |  |  |  |
| t(4; 14) |  |  | 0.000 |  |  |  |  |
| ASCT | 0.426 | 0.293-0.620 | 0.017 |  | 0.403 | 0.225-0.720 | 0.002 |
| Abbreviations: PT: prothrombin time; APTT: activated partial thromboplastin time; IMiD: immunomodulatory; ASCT: autologous stem cell transplant | | | | | | | |

| Supplementary table 2. Best response rate after induction therapy in all patients | | | |
| --- | --- | --- | --- |
|  | Overall  n=324  n (%) | lengthened  PT or APTT  n=138  n (%) | normal  PT and APTT  n=186  n (%) |
| sCR | 64(19.8) | 25(18.1) | 39(21.0) |
| CR | 53(16.4) | 17(12.3) | 36(19.4) |
| VGPR | 72(22.2) | 32(23.2) | 40(21.5) |
| PR | 91(28.1) | 39(28.3) | 52(28.0) |
| SD | 31(9.6) | 15(10.9) | 16(8.6) |
| PD | 13(4.0) | 10(7.2) | 3(1.6) |
| Abbreviations: PT, prothrombin time; APTT, activated partial thromboplastin time; sCR, stringent complete response; CR, complete response; VGPR, very good partial response; PR, partial response; SD, stable disease; PD, progressive disease. | | | |

| Supplementary table 3. Best response rate after induction therapy in matched patients | | | |
| --- | --- | --- | --- |
|  | Overall  n=154  n (%) | lengthened  PT or APTT  n=77  n (%) | normal  PT and APTT  n=77  n (%) |
| sCR | 33(21.4) | 17(22.1) | 16(20.8) |
| CR | 24(15.6) | 10(13.0) | 14(18.2) |
| VGPR | 37(24.0) | 17(22.1) | 20(26.0) |
| PR | 35(22.7) | 18(23.4) | 17(22.1) |
| SD | 18(11.7) | 9(11.7) | 9(11.7) |
| PD | 7(4.5) | 6(7.8) | 1(1.3) |
| Abbreviations: PT, prothrombin time; APTT, activated partial thromboplastin time; sCR, stringent complete response; CR, complete response; VGPR, very good partial response; PR, partial response; SD, stable disease; PD, progressive disease. | | | |
